# Comprehensive analysis of the lysine acetylome in *Aeromonas hydrophila* reveals cross-talk between lysine acetylation and succinylation in LuxS

**DOI:** 10.1080/22221751.2019.1656549

**Published:** 2019-08-26

**Authors:** Lina Sun, Zujie Yao, Zhuang Guo, Lishan Zhang, Yuqian Wang, Ranran Mao, Yuexu Lin, Yuying Fu, Xiangmin Lin

**Affiliations:** a Fujian Provincial Key Laboratory of Agroecological Processing and Safety Monitoring, School of Life Sciences, Fujian Agriculture and Forestry University, Fuzhou, People’s Republic of China; b Key Laboratory of Crop Ecology and Molecular Physiology, Fujian Agriculture and Forestry University, Fujian Province University, Fuzhou, People’s Republic of China; c Shanghai Key Laboratory of Plant Functional Genomics and Resources, Shanghai Chenshan Plant Science Research Center, Chinese Academy of Sciences, Shanghai, People’s Republic of China; d Key Laboratory of Marine Biotechnology of Fujian Province, Institute of Oceanology, Fujian Agriculture and Forestry University, Fuzhou, People’s Republic of China

**Keywords:** Lysine acetylome, *Aeromonas hydrophila*, cross-talk, LuxS, lysine succinylome

## Abstract

Lysine acetylation and succinylation are both prevalent protein post-translational modifications (PTMs) in bacteria species, whereas the effect of the cross-talk between both PTMs on bacterial biological function remains largely unknown. Our previously study found lysine succinylated sites on proteins play important role on metabolic pathways in fish pathogenic *Aeromonas hydrophila*. A total of 3189 lysine-acetylation sites were further identified on 1013 proteins of this pathogen using LC-MS/MS in this study. Functional examination of these PTMs peptides showed associations with basal biological processes, especially metabolic pathways. Additionally, when comparing the obtained lysine acetylome to a previously obtained lysine succinylome, 1198 sites in a total of 547 proteins were found to be in common and associated with various metabolic pathways. As the autoinducer-2 (AI-2) synthase involved in quorum sensing of bacteria, the site-directed mutagenesis of LuxS at the K165 site was performed and revealed that the cross-talk between lysine acetylation and succinylation exerts an inverse influence on bacterial quorum sensing and on LuxS enzymatic activity. In summary, this study provides an in-depth *A. hydrophila* lysine acetylome profile and for the first time reveals the role of cross-talk between lysine acetylation and succinylation, and its potential impact on bacterial physiological functions.

## Introduction

During the regulation of several key biological processes, protein post-translational modifications (PTMs), such as acylation, phosphorylation, and methylation, reversibly modify specific amino acid residues and play important roles in maintaining diverse cellular processes and functions [1,2]. Of these PTM sites, lysine is the most commonly modified amino acid residue, with only a single ionic interaction formed, thus offering more functional flexibility following acetylation, methylation, or ubiquitination [3]. Of these potential modifications, lysine acetylation is one of the most common [4]. Numerous studies have reported that this modification is widespread in both eukaryotes and prokaryotes and participates in the regulation of multiple complicated metabolic pathways [5]. In prokaryotic organisms, thousands of lysine acetylated modification sites have been identified in *Escherichia coli*, *Mycobacterium tuberculosis* and *Pseudomonas aeruginosa* in recent years [6–8]. Furthermore, this modification has been associated with virulence, chemotaxis, and stress responses, in addition to enzyme activity regulation [9,10]. However, despite all of these associated roles, the role of acetylation in many intrinsic biological functions still remains elusive.

*Aeromonas hydrophila* is a Gram-negative, heterotrophic bacteria, and is also a well characterized pathogen that predominantly infects freshwater fish and is an opportunistic pathogen in humans and other animals. In recent years, human infections by this pathogen are increasing due to its severe antibiotic resistance, thus making the infection potentially life threatening [11,12]. Although there are many researches currently focusing on the virulence, stress-resistant mechanism or the vaccine development, the cellular physiology and pathology of *A. hydrophila* are still largely unknown [13–15]. Thus, based on the facts that PTMs play an important role in the regulation of diverse biological functions, a better understanding of bacterial PTMs is necessary to better characterize this organism. In our previous study, lysine succinylation, a novel lysine PTM, was investigated in *A. hydrophila* ATCC 7966 and a total of 2174 succinylated modification sites were identified [16]. Further analysis indicated that the modifications are involved in several essential physiological processes, including energy metabolism pathway, TCA cycle, and protein export. In other studies, some bacterial species have been shown to have succinylated or acetylated lysine residues at the same PTM sites as well, thus suggesting that both PTMs may co-regulate some key biological pathways in an unknown manner [17]. However, the physiological functions associated with lysine acetylation and whether any lysine cross-talk with succinylation is present in bacterial species remains elusive.

In the present study, we firstly provided a comprehensive lysine acetylome profile for *A. hydrophila* ATCC7966 by combining high immune-affinity enrichment with high-resolution liquid chromatography-tandem mass spectrometry (LC-MS/MS) analysis. The identified acetylated proteins and adopting motifs were then further elucidated by performing Gene Ontology (GO) and KEGG (Kyoto Encyclopedia of Genes and Genomes) enrichment analyses. Moreover, several of the acetylated proteins in the current work were confirmed by co-immunoprecipitation integrated with Western blot analysis. Next, a comparative analysis with the lysine succinylome revealed that acetylation and succinylation modulation is correlated within the cell. To explore the lysine cross-talk between acetylation and succinylation and its relationship to bacterial biological processes, site-directed mutagenesis of S-ribosylhomocysteine lyase (LuxS) at the K165 site was performed, and the subsequent impact on autoinducer 2 (AI-2) production and fitness in *A. hydrophila* was examined. Additionally, an enzyme activity assay was performed to further elucidate the potential impact of acetylation and cross-talk in LuxS*.* Overall, this study provides a promising approach to further explore lysine acetylation and cross-talk with succinylation and their functional impact on physiological processes in *A. hydrophila*.

## Materials and methods

Overnight propagated *A. hydrophila* ATCC 7966 was diluted 1:100 into 200 ml Luria–Bertani (LB) medium at 30°C and harvested when an OD_600nm_ of 1.0 was reached. After sonicated on ice for a total of 20 min, the protein solutions were reduced with DTT and alkylated with iodoacetamide (IAA) and then digested by trypsin at a ratio of 1:50 overnight at 37°C [18]. The lysine-acetylated peptides were enriched using a PTMScan Acetyl-Lysine Motif (Ac-K) Kit (Cell Signal Technology, Danvers, MA, USA) and identified via MS on a Q Exactive mass spectrometer (Thermo Fisher Scientific). The raw data files obtained from the MS analysis were processed using MaxQuant with an integrated Andromeda search engine (v.1.4.1.2) with the false discovery rate (FDR) <0.01; the identified lysine acetylation sites with a localization probability >0.75. The UniProt-GOA database, KAAS tool, and InterPro domain database were used for bioinformatics analysis [19,20]. Several candidate proteins were selected to validate their lysine acetylated status by Co-IP and western blotting as previously described [21–23]. The site-directed mutagenesis of *luxS* (K165E, K165Q and K165R) was performed with the corresponding primers (Table S2) using the Fast Mutagenesis System Kit and inserted in the broad host vector His-tagged pBBR1-MCS1 [13,16,24]. The AI-2 activity in the *luxS* mutant strains were analysed using the *Vibrio harveyi* BB170 bioluminescence reporter strain as previously described [25]. Purified LuxS protein was in vitro acetylated and succinylated and the enzyme activities were detected as previously described [4,26–29]. Bacterial competitive ability assays between *A. hydrophila* and *Vibrio alginolyticus* were conducted, as previously described with some modifications [30]. Finally, we also tested the enzyme activity of site-specific acetyllysine modified recombinant LuxS protein using a two-plasmid system with slightly modifications [4,31–33]. For full details of all these processes, please see SI Appendix and SII Appendix.

## Results

### Systematic identification of lysine acetylome in *A. hydrophila*

**Figure 1.**
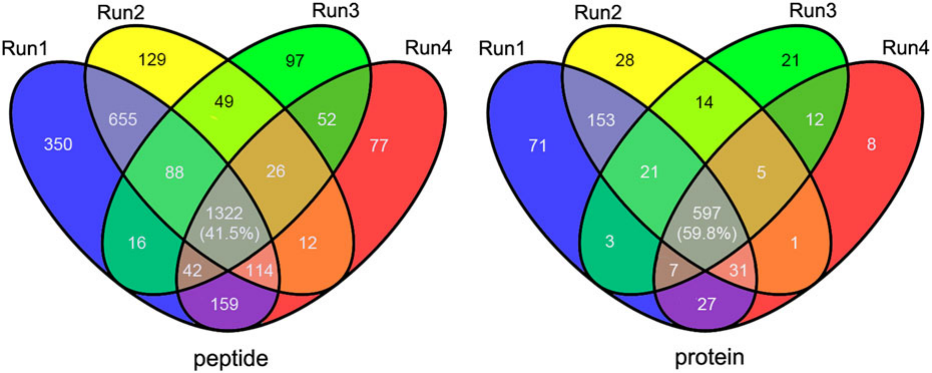
Overlap between the acetylated peptides and proteins from four replicates in *A. hydrophila*. (A and B) Venn diagram showing the numbers of identified acetylated peptides and proteins, respectively.

In this study, a proteomics approach was integrated with immune-affinity purification to enrich lysine acetylated peptides in *A. hydrophila* that were then analysed via high-resolution LC-MS/MS. To ensure the reliability of the date, four independent replicates were run and an observed modification was required to be present in at least two of the four runs to be considered an acylation site. As showed in Figure 1, many acetylated identified proteins (>150) are present in run 1 and 2 but absent from replicates 3 and 4, and the intersection between all replicates at the protein level and peptide levels is only ∼40 and ∼60%, respectively. That may largely come from the experimental errors during immunoaffinity enriching procedure, indicating the importance of biological replicates for more accurate results. Therefore, a total of 3189 lysine acetylated peptides matching 1013 unique proteins were identified (Table S1), which accounted for 24.6% (1013/4123) of the total proteins that were identified in *A. hydrophila*.

### Functional annotation of the lysine acetylome in *A. hydrophila*

**Figure 2.**
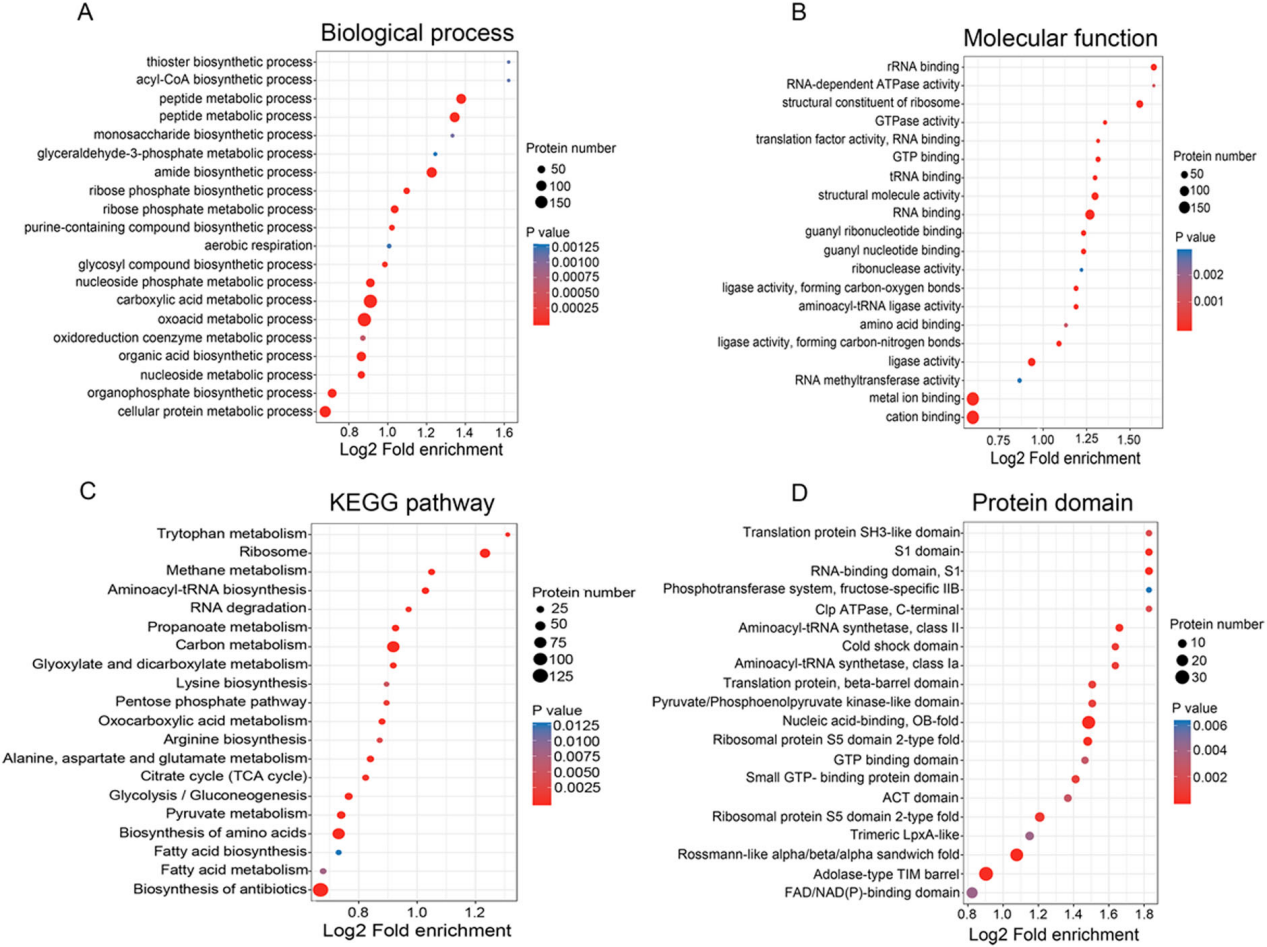
Bubble plot showing the enrichment for GO, KEGG pathway, and Pfam domain of acetyl-proteins. (A and B) GO classification of the identified acetylated proteins in terms of biological process and molecular function. (C) KEGG pathway-based enrichment analysis of acetylated proteins. (D) Pfam domain enrichment analysis of the identified proteins.

To further examine the identified acetylated sites, GO, KEGG and Pfam domain analyses were performed. Accordingly, the bubble plots were constructed to visualize the different proteins that are associated with the acetylated sites (Figure 2), in order to determine their functions and associated pathways. For the GO annotations in the biological process category (Figure 2(A)), the lysine modified proteins were mainly annotated to the categories of the oxoacid, cellular protein and carboxylic acid metabolic process. In the molecular function category, the identified acetyl proteins tended to be associated with metal ion binding, structural constituent of ribosome, RNA binding and ligase activity, and so on (Figure 2(B)). Enrichment analysis of KEGG pathway analysis demonstrated that these acetylated proteins were significantly enriched in carbon metabolism, ribosome, and biosynthesis of amino acids, suggesting that the lysine acetylation proteins were mainly involved in diverse metabolic pathways and translation (Figure 2(C)). Furthermore, the enrichment analysis of functional protein domain displayed that the lysine acetylated residues were mainly located in the specific domains such as nucleic acid-binding, RNA-binding domain, translation protein and aminoacyl-tRNA synthetase (Figure 2(D)). Overall, these results implied that the protein acetylation may exert a regulatory role in a variety of essential cellular processes such as metabolism, translation and biosynthesis in *A. hydrophilia*.

### Functional interaction network for the acetylated proteins

**Figure 3.**
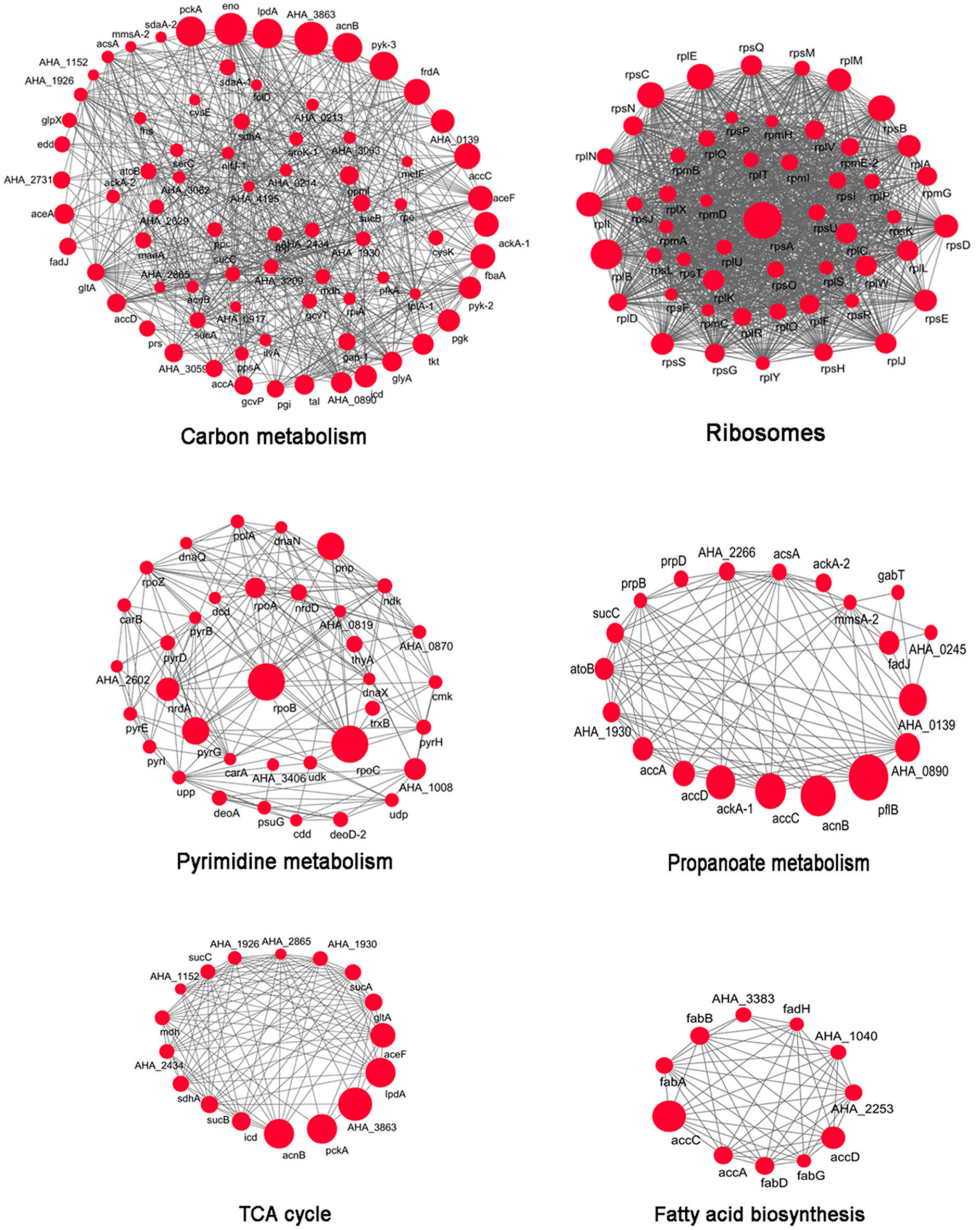
Protein–protein interaction networks in acetylated proteins. Six highly interconnected sub-networks in acetylated proteins were showed. They are carbon metabolism, ribosome, TCA cycle, pyrimidine metabolism, propanoate metabolism and fatty acid biosynthesis. The size of each circle reflects the number of acetylation sites.

To obtain a more in-depth picture of PTM proteins’ function, it is necessary to consider its multifarious functional partnerships and protein interactions [34]. Investigating these protein interactions provides a detailed view of complicated cellular processes and the systematic regulation of numerous biological functions [35,36]. Thus, to examine these interactions based on the *A. hydrophila* acetylome, protein–protein interaction networks were established based on the STRING database using Cytoscape [37,38]. For each protein interaction network, the acetylated proteins associated with that pathway and an overview of how any one of protein functions *in vivo* is provided. By using this approach, six highly interconnected sub-networks were identified (Figure 3). Among of them, 76 acetyl proteins were found to be associated with carbon metabolism and comprised the most tight-ranking connection cluster. Meanwhile, the remaining five proteins were associated with ribosome, pyrimidine metabolism, propanoate metabolism, TCA cycle, and fatty acid biosynthesis pathways. In previous studies in prokaryotes, similar interaction networks involving ribosome and TCA cycle pathways were also noted [7]. Taken together, these results suggest that lysine acetylation plays a pivotal role in translation and the TCA cycle in *A. hydrophila.* These findings also suggest that the conservation of lysine acetylation may be due to its involvement in a wide range of regulatory mechanisms and that it may function in diverse cellular processes in *A. hydrophila* and even other prokaryotes [39].

### Acetylome verification with co-immunoprecipitation (Co-IP) coupled with Western blotting analysis

**Figure 4.**
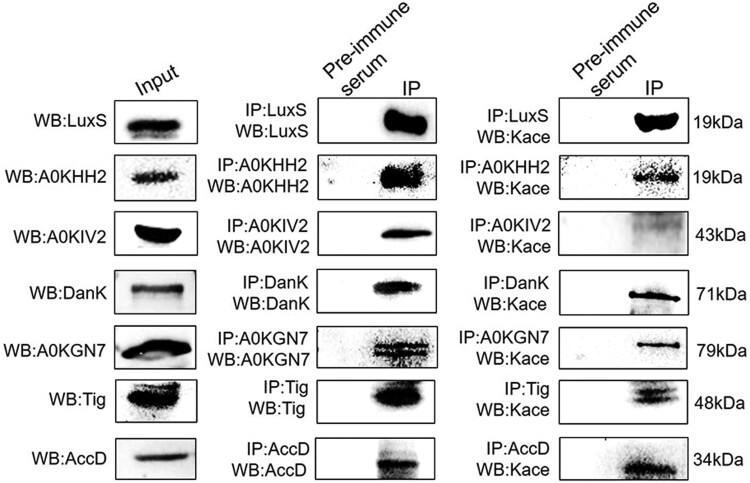
Validation by combining immunoprecipitation with Western blotting using anti-acetyl lysine antibodies. Left panel: Western blot analysis of target nine proteins. Middle panel: Western blot analysis for the Co-IP of whole-cell proteins with rabbit antisera to the nine proteins serving as a primary antibody and pre-immune serum used as the normal IgG. Right panel: Western blot of Co-IP samples using primary anti-acetyl lysine antibodies. The Kace represents lysine acetylation.

To confirm the lysine acetylome results, seven acetylated proteins (LuxS, A0KHH2, A0KIV2, DnaK, A0KGN7, Tig, and AccD) were examined using Co-IP with Western blotting. The target proteins were enriched using custom specific antibodies and visualized via Western blotting using anti-target proteins or anti-acetyl lysine rabbit polyclonal primary antibodies, with normal Western blotting against the target proteins as a positive control. The normal Western blotting that utilized *A. hydrophila* whole cell proteins displayed a high specificity for their antibodies of all target proteins (Figure 4). Further analysis also indicates that these nine antibodies successfully enriched the target proteins after the Co-IP procedure, with pre-immune serum used as a negative control. Finally, lysine acetylation was successfully detected in the target proteins when using primary anti-acetyl antibodies in the immunoprecipitated samples, thus indicating that the acetylome results are reliable.

### Overlap between lysine acetylation and succinylation in *A. hydrophila*

**Figure 5.**
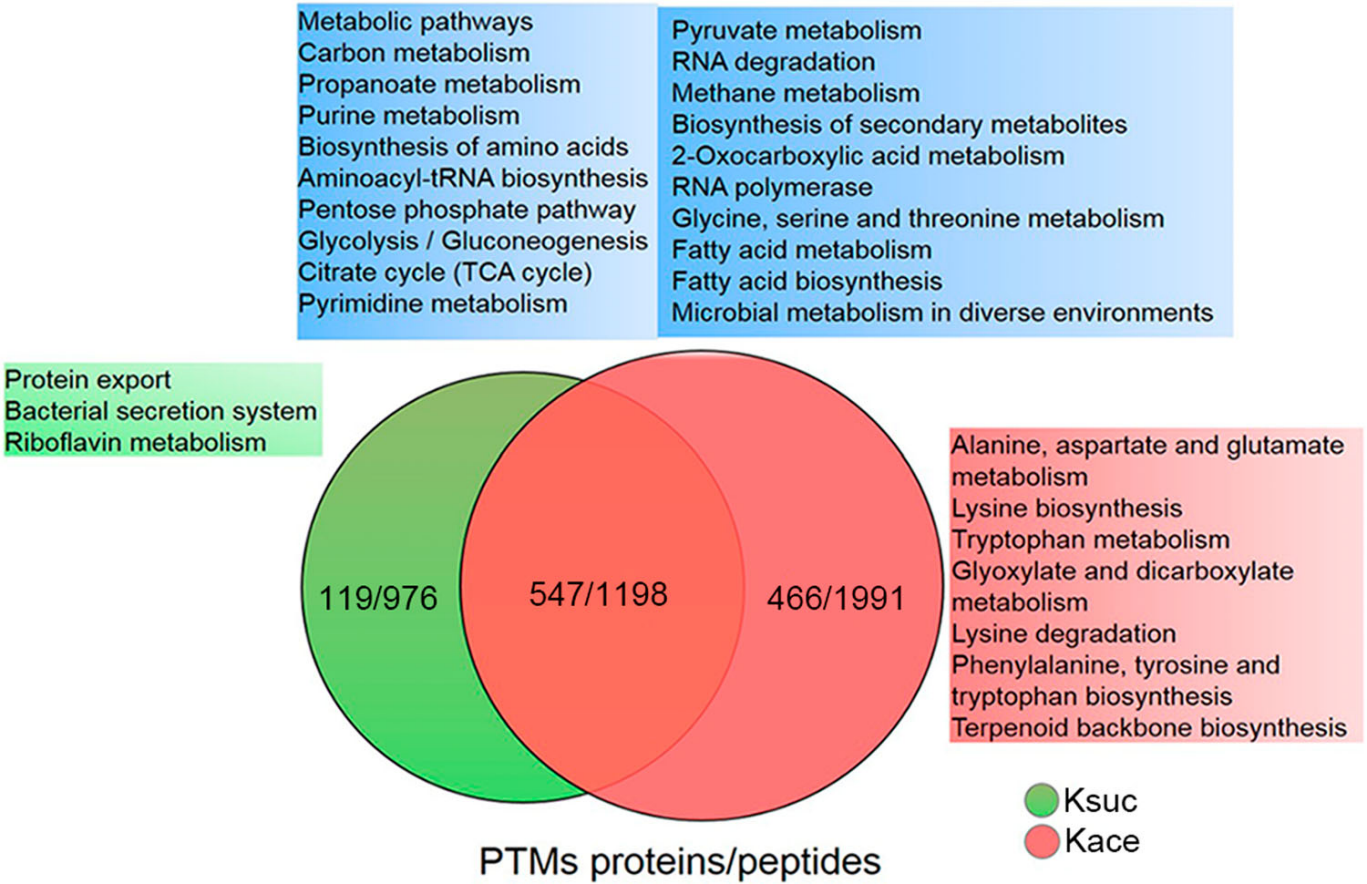
The KEGG pathway overlap between the lysine acetylome and succinylome in *A. hydrophila*. Venn diagram displaying the overlap number of peptides and proteins between both PTMs. The blue, green and red area shows the KEGG pathways associated with the overlapping, unique succinylated and unique acetylated proteins, respectively. The Kace and Ksuc represent lysine acetylation and succinylation, respectively.

Various studies have shown that lysine residues are preferential modification site for various kinds of PTMs, such as acetylation, succinylation, malonylation, glutarylation, methylation, and ubiquitination [3]. In our previous study, we examined the lysine succinylome in *A. hydrophila* and identified 2174 lysine succinylation modification sites within 666 proteins [16]. Hence, to observe any potential cross-talk between lysine acetylation and succinylation*,* the acetylation modification characteristics were compared with the *A. hydrophila* succinylome and identified 1198 peptides comprising 547 proteins that were overlapped (Figure 5). The extensive overlap between two types of PTMs implied that succinylation and acetylation frequently occurred at the same lysine residues in *A. hydrophila*. Furthermore, KEGG pathway analysis showed that most of the overlapping proteins are associated with carbon metabolism, glycolysis/gluconeogenesis, TCA cycle, and pyruvate metabolism. Moreover, several processes, such as protein export, the bacterial secretion system, and riboflavin metabolism, were found to be unique to lysine succinylation, whereas alanine, aspartate, and glutamate metabolism, lysine biosynthesis, glyoxylate and dicarboxylate metabolism were unique to acetylation*.* These results indicate that the physiological functions of these two PTMs may have their own special features, which will require further investigation.

**Figure 6.**
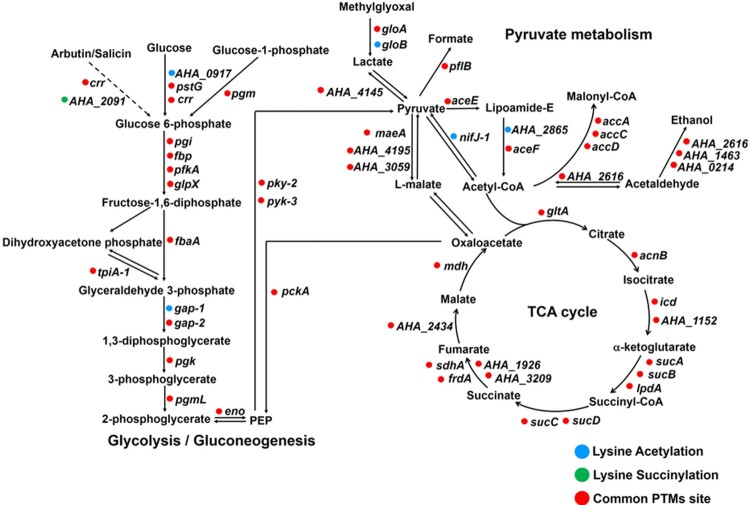
Schematic representation of lysine acetylation and succinylation in glycolysis/gluconeogenesis, TCA cycle, and pyruvate metabolism. The circle colour represents acetylation (blue), succinylation (green), or common PTMs sites (red), respectively.

Moreover, central metabolic pathways, such as glycolysis/gluconeogenesis, TCA cycle and pyruvate metabolism, were examined for both of the lysine modifications. The results showed that most of the PTM sites were modified by both acetylation and succinylation (Figure 6). Only one proteins, A0KK20 (beta-glucosidase, *AHA_2091*) had only succinylation, while five proteins were only acetylated including A0KGR4 (polyphosphate glucokinase, *AHA_0917*), A0KIK2 (hydroxyacylglutathione hydrolase, *gloB*), A0KNJ9 (glyceraldehyde-3-phosphate dehydrogenase, *gap-1*), A0KM68 (dihydrolipoamide acetyltransferase component of pyruvate dehydrogenase complex, *AHA_2865*), and A0KID4 (pyruvate-flavodoxin oxidoreductase, *nifJ-1*). Overall, these findings demonstrate that lysine acetylation and succinylation are both significantly enriched and broadly distributed in central metabolic pathways, thus implying that both types of modification may “cross-talk” when modulating complicated intracellular pathways [40].

### Lysine acetylation cross-talk with succinylation in LuxS at the K165 Site

**Figure 7.**
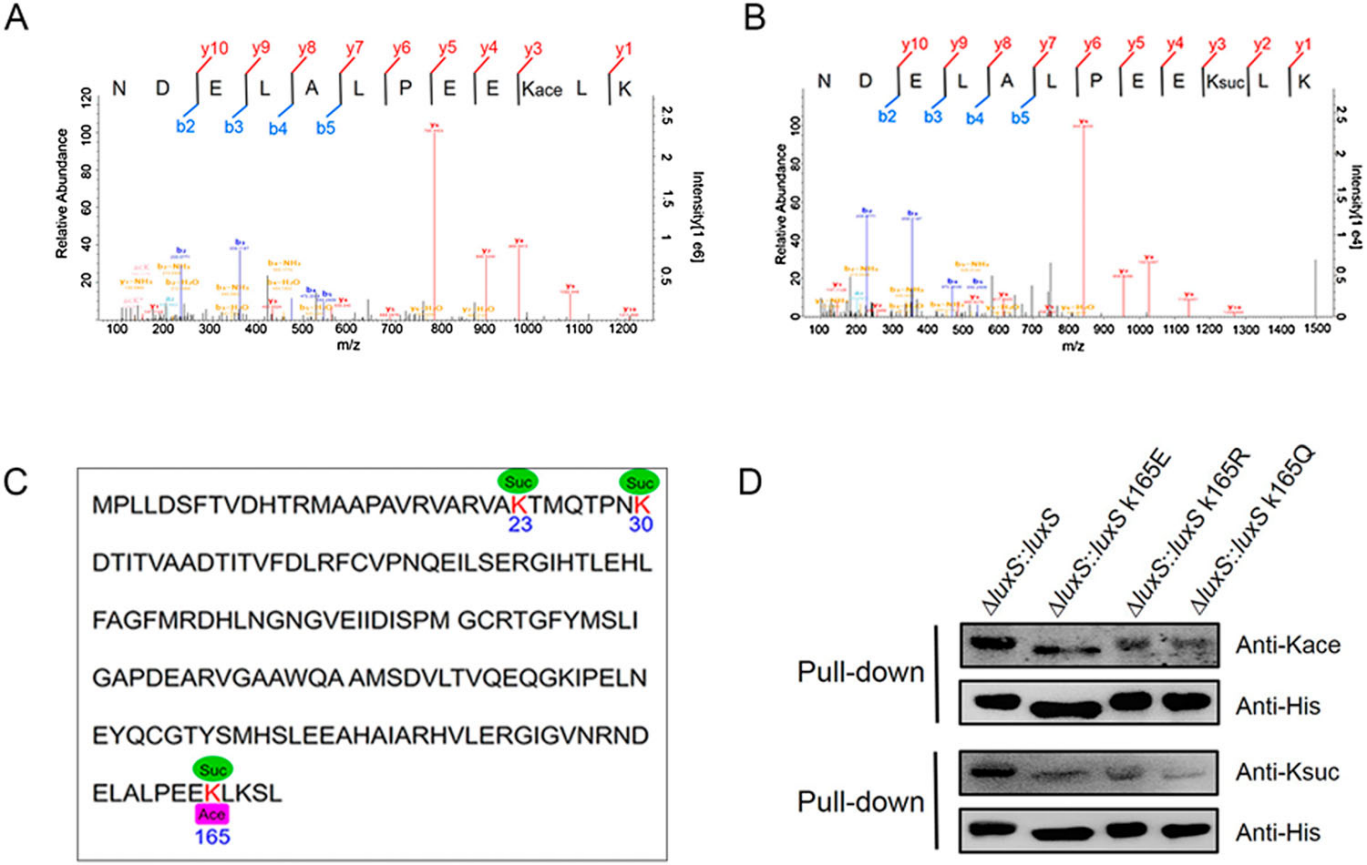
Lysine cross-talk between acetylation and succinylation at the K165 site of LuxS. (A) and (B) MS/MS spectra of acetyl and succinyl-peptides for the LuxS K165 site in *A. hydrophila*. (C) Lysine acetylation and succinylation modifications on LuxS sequence in *A. hydrophila*. (D) The LuxS site K165 PTMs in the rescued strain and site-directed mutants as detected by Western blotting combined with a pull-down assay using anti-acetyl and anti-succinyl antibodies, respectively. Kace, lysine acetylation; Ksuc, lysine succinylation.

The lysine acetylome results found that LuxS is acetylated at the K165 site (Figure 7(A)). Interestingly, the present study showed that acetylated LuxS directly cross-talks with succinylation at the K165 site in *A. hydrophila* ATCC 7966 (Figure 7(B)). In addition, our previously research also showed that the K23 and K30 sites on LuxS are succinylated as well, and both are likely to positively regulate its enzymatic function (Figure 7(C)) [16]. However, if the cross-talk of K165 site between acetylation and succinylation affects biological function or not is still unknown.

To test this possibility, the LuxS K165 site was mutated to glutamate (E), arginine (R), or glutamine (Q) to mimic lysine succinylation, deacylation, and acetylation modifications, respectively. Subsequently, 6xHis-tag site-directed mutagenesis plasmids were constructed and a *ΔluxS* strain was generated. Each mutant strain contained a site-directed mutation at K165E, K165R or K165Q and was complemented with K165 as a control. A pull-down assay was performed and evaluated via Western blotting using anti-acetyl and anti-succinyl antibodies, respectively. The results showed that all of the point mutations led to a decrease in acetylation and succinylation, thus indicating that the K165 site did have both modifications (Figure 7(D)).

### Lysine cross-talk occurs on LuxS and affects LuxS enzyme activity

**Figure 8.**
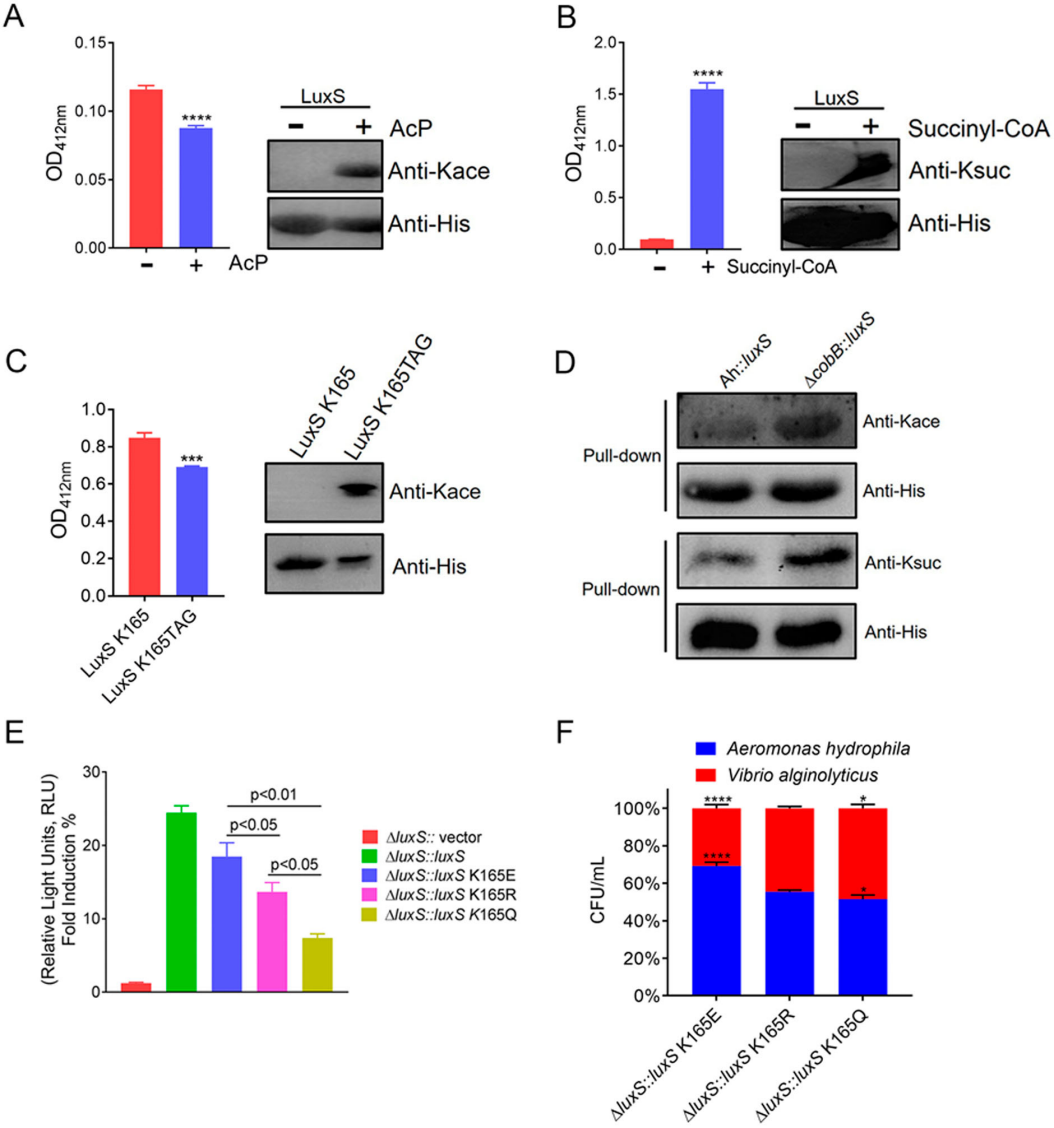
The PTMs cross-talk in LuxS influences its physiological functions and acylated by CobB deacetylase. (A) and (B) The enzymatic activity of the recombinant LuxS with *in vitro* acetylation and succinylation, and Western blot analysis using anti-acetyl lysine and anti-succinyl lysine, respectively. (C) The effect of site-specific acetylation occurred at K165 site of LuxS protein on the LuxS enzyme activity. Acetylation level of the site-specific acetylation occurred on K165TAG site of recombinant LuxS protein in *E. coli* was determined by Western blotting and the effect of the acetylated K165TAG site on LuxS enzyme was estimated by the enzyme activity. (D) The pull-down assay combined with Western blotting was utilized to detect CobB deacetylation and desuccinylation activity. Herein, *Ah* indicated the wild-type strain. (E) AI-2 production in the *luxS* mutant strains were determined using the *V. harveyi* bioluminescence assay. (F) *A. hydrophila* LuxS site-directed mutagenesis and its effect on bacterial fitness when competing with *V. alginolyticus*. Statistical analysis was performed using the One-way ANOVA, with each *p*-value showed in the bar graph. The Kace and Ksuc denote lysine acetylation and succinylation, respectively.

To gain a further insight into the biological function of lysine cross-talk between acetylation and succinylation on AI-2 synthase, LuxS enzyme activity was monitored using S-ribosyl-L-homocysteine as the reacting substrate in *vitro*. Purified recombinant LuxS was acetylated (AcP) or succinylated (succinyl-CoA) and then allowed to react with the substrate while monitoring the activity levels at an absorbance of 412 nm. Western blot analysis further confirmed that these proteins were acetylated or succinylated *in vitro*. The results showed that the AcP-LuxS had a lower enzymatic activity when compared to those without acetylation (Figure 8(A)), whereas the succinylated LuxS enzyme showed a significantly enhanced enzymatic activity (Figure 8(B)). Moreover, we further purified K165 site-specific acetylated recombinant His-tag LuxS in *E. coli* using a two-plasmid-based system of genetically encoding N^Ɛ^-acetyllysine. The following enzymatic activity assay displayed the similar results as the conclusions *in vitro* (Figure 8(C)). These results reveal that acetylation may negatively regulate LuxS enzymatic activity in *A. hydrophila*, while succinylation may act as a positive regulator.

### Cobb catalyses lysine deacetylation and desuccinylation on LuxS

The CobB, a bifunctional enzyme that can catalyse the lysine deacetylation and desuccinylation, belonged to the Sir2 family and is a common sirtuin in bacteria [41]. To evaluate the deacetylase or desuccinylase activity of CobB on the lysine modified LuxS, we conducted the deacetylation and desuccinylation assay *in vivo*. The pull-down assays of both complemented variants, including Δ*cobB*::*luxS* and *A. hydrophila*::*luxS*, were performed and Western blotting was used for detecting the deacetylase and desuccinylase activity with anti-acetyl and anti-succinyl antibodies, respectively. The results showed that the loss of CobB could moderately increase the acetylation and succinylation modification on LuxS (Figure 8(D)), which suggest that CobB is a bifunctional protein participating in both lysine acetylation and succinylation elimination on LuxS.

### The effect of lysine cross-talk occurring on LuxS K165 on AI-2 activity of *A. hydrophila*

The signal molecule AI-2 is produced by the enzyme LuxS catalysing the precursor S-ribosyl-L-homocysteine. To further investigate the influence of lysine cross-talk on the LuxS enzyme activity, the relative level of AI-2 produced in each *luxS* derivative was measured using *V. harveyi* BB170 bioassay. The exogenous AI-2 in cell-free culture fluids of test strains is capable to induce bioluminescence produced by the reporter strain BB170, and AI-2 activity was expressed as relative light units (RLU) obtained by the ratio the resulting light production of each sample to positive control. The obtained rescued strain exhibited significantly higher AI-2 activity levels when compared to all of the site-directed mutants. Furthermore, the AI-2 production in the K165Q mutant was substantially decreased when compared to the K165E and K165R mutants (Figure 8(E)). These results indicate that lysine cross-talk is affected by both modifications on the LuxS K165 site and has a remarkable impact on AI-2 activity levels.

### The lysine cross-talk of LuxS K165 site in *A. hydrophila* affects competition with other bacterial species

Since LuxS works on the bacterial AI-2 type quorum sensing, the lysine cross-talk on this protein may affect the fitness of *A. hydrophila* when communicating with other bacterial species. To test this, the *luxS* mutants were co-cultured with *V. alginolyticus*, and colony numbers were counted. As showed in Figure 8(F), the bacterial numbers of the mimic-acetylated K165Q mutant were lower than those of the non-acetylated K165R strain, while the mimic-succinylated K165E was higher. These findings suggest that lysine cross-talk between both PTMs is likely to play a positive role in modulating communication between *A. hydrophila* and other bacteria, and that just lysine acetylation on K165 might be a negative regulator, while succinylation acts as a positive regulator. The enzyme activity at LuxS K165 site displayed a similar tendency as the previous report at K23 and K30 sites, thus indicating that the biological functions of LuxS may be precisely regulated by multiply PTMs [16].

## Discussion

At present, more than 300 different types of PTMs are known to occur at various time points [1,42]. PTMs strongly impact protein properties by modulating protein activity levels via the addition of a modifying chemical group or another protein to a specific amino acid residue within the protein [43]. Moreover, lysine acetylation, which is one of the most abundant and evolutionarily conserved post-translational modifications, is also thought to exert a pivotal influence on bacterial physiology in various ways [5]. However, little is known regarding this PTM in *A. hydrophila*.

In the present work, the *A. hydrophila* ATCC 7966 lysine-acetylated proteome at the middle logarithmic phase was comprehensively investigated by utilizing high-affinity peptide enrichment coupled with high-resolution MS. At the middle logarithmic phase, the physiologic functions of bacteria are extremely dynamic in vivo, which contributes to observing the regulatory role of lysine acetylation for a series of cellular functions of *A. hydrophila*. Results displayed that a total of 3189 lysine-acetylated peptides matched to 1013 unique acetylated proteins were identified and associated with a broad spectrum of essential cellular pathways ranging from multifarious metabolic processes to translation processes. The further bioinformatics analysis showed that these acetylated proteins are extensively involved in diverse physiological processes. Like *A. hydrophilia*, the similar findings have been uncovered in other bacteria such as *Streptococcus pneumoniae*, *M. tuberculosis* and *P. aeruginosa*, suggesting that the regulatory functions based on protein acetylation might be a conservative regulatory mechanism in prokaryotes [7,8,44].

Moreover, the obtained acetylome was compared to a previously obtained *A. hydrophila* succinylome profile, and 547 acetyl proteins and 1198 acetylated sites were found to also be succinylated [16]. Therefore, it is a problem worthy of in-depth study and exploration on the importance and relevance of both PTMs at the same lysine residues. Our further functional characterization showed that both PTMs may cross-talk to modulate some key intracellular metabolic pathways such as TCA cycle and glycolysis/gluconeogenesis.

To further investigate the role of cross-talk between both PTMs, the K165 site of S-ribosylhomocysteine lyase LuxS is take as an example for analysing the effect of both PTMs on biological functions. LuxS is a key enzyme in the production of quorum sensing (QS) molecules and plays an important role in signaling communication with other bacterial species [45]. Moreover, it is a key regulatory enzyme in the activated methyl cycle pathway that modulates methyl donor production and is involved in sulfur metabolism [46,47]. We firstly validated that LuxS was modified by acetylation and succinylation at K165 site and deacetylated and desuccinylated by CobB Western blotting in this study. Additionally, CobB in *E. coli* displays limited deacetylase activity, which suggests that additional lysine deacetylases (KDACs) may exist [48]. To test this possibility, we also tested the lysine deacetylated and desuccinylated activities in A0KI27 protein (gene name *AHA_1389*), which belongs to the CobB family in *A. hydrophila*. However, we can’t find clear related enzymatic activities in this homologue (data not show), indicating the intrinsic mechanism should be further investigated in the future. Moreover, A closer examination of the cross-talk between acetylation and succinylation at the LuxS K165 site showed that it modulates AI-2 activity and can interfere with bacterial QS as well as influence bacterial communication with other species. Additionally, an enzyme activity assay demonstrated that lysine acetylation negatively modulates LuxS enzymatic activity, while succinylation acts as a positive regulator. These findings strongly suggest that lysine cross-talk is important for modulating protein function.

In addition to the metabolic process, nowadays the increasing studies have displayed that lysine acetylation possibly exerts a significant influence on regulating the virulence of lots of pathogens, such as *S. pneumoniae*, *P. aeruginosa* and *S. typhimurium* [8,44,49]. As a common pathogen in aquatic surroundings, the lysine acetylation was also observed on several described virulence factors of *A. hydrophila*, such as Fur (A0KIG7, ferric uptake regulation protein), AhyR (A0KFR3, acyl-homoserine-lactone synthase), CheY (A0KI21, chemotaxis protein), CheA (A0KI23, chemotaxis protein), Eno (A0KGH3, enolase). These proteins play important roles on various virulence related processes, including iron homeostasis, quorum sensing or be as virulence factor directly [50–52]. These results further imply that protein acetylation involves in the pathogenesis of various kinds of bacteria. Taken together, this research provides an important foundation for investigating the biological functions and regulatory roles of lysine acetylation and lysine cross-talk with succinylation in *A. hydrophila*.

## Supplementary Material

Supplemental Material
