# Supplementary material for: Comprehensive analysis of the lysine acetylome in Aeromonas hydrophila reveals cross-talk between lysine acetylation and succinylation in LuxS
Source: Emerg Microbes Infect. 2019 Aug 26;8(1):1229–39. doi: 10.1080/22221751.2019.1656549 (PMC6735345; doi:10.1080/22221751.2019.1656549)
Supplement: Supplemental Material [file TEMI_A_1656549_SM0185.zip › SI Appendix - EMI_final version_20190714v1.docx]

**Materials and methods**

**Protein extraction and in-solution trypsin digestion**

*A. hydrophila* ATCC 7966 was propagated overnight in 5 ml of Luria-Bertani (LB) medium at 30°C, and then diluted 1:100 into 200 ml of fresh medium for further expansion. The cultured cells were harvested by centrifugation at 14,000 x g for 20 min at 4°C when *A. hydrophila* was grown at the middle logarithmic growth (OD_600nm_ = 1.0). The growth curve of *A. hydrophila* ATCC 7966 was presented in **Figure S1** of **SII Appendix**. The bacterial pellets were then washed twice with cold phosphate buffered saline (PBS, pH 7.4), resuspended in chilled lysis buffer [8 M urea, 2 mM EDTA, dithiothreitol (DTT), 1% protease inhibitor cocktail, 3 μM trichostatin A, and 50 mM nicotinamide] and sonicated on ice for a total of 20 min at 9-s intervals [[1](#_ENREF_1)]. Unbroken cells and cellular debris were removed via centrifugation at 20,000 x g for 10 min at 4°C and the supernatant was filtered (0.22 µm) prior to quantification using a BCA Protein Assay Kit (Thermo Fisher Scientific, Rockford, IL, USA).

For peptide digestion, the protein solutions were first reduced with 10 mM DTT for 1 h at 37°C and alkylated for 45 min with 20 mM iodoacetamide (IAA) at room temperature in the dark. The samples were then diluted to obtain a <2 M urea concentration with 100 mM (NH_4_)_2_CO_3_. Next, the trypsin was added at a ratio of 1:50 and the samples were digested overnight at 37°C. Finally, the digested peptides were dried down using a CentriVap concentrator (Labconco Inc., Kansas City, MO, USA).

**Affinity enrichment of lysine-acetylated peptides**

Freeze-dried samples were dissolved in NETN buﬀer (100 mM NaCl, 1 mM EDTA, 50 mM Tris-HCl, 0.5% NP-40; pH 8.0), and pre-washed with Anti-Ac-K antibody beads using a PTMScan Acetyl-Lysine Motif (Ac-K) Kit (Cell Signal Technology, Danvers, MA, USA) and incubated at 4°C for 1.5 h with gentle shaking. The beads were then washed at least three times with pre-cooled NETN buffer and pre-cooled water. The bound peptides were eluted twice with 40 μl 0.15% TFA at room temperature for 10 min per elucidation. The eluted peptides were then pooled and desalted by using a Sep-Pak SPE C18 (Waters Inc. Milford, MA, USA) and dried down as before.

**Lysine-acetylation peptide identification via LC-MS/MS**

The peptide mixture, which contained 0.1% formic acid (FA), was loaded onto a reverse phase trap column (Acclaim PepMap100, 100 μm × 2 cm, nanoViper C18; Thermo Scientific) connected to a C18-reversed phase analytical column (Easy Column: length, 10 cm; i.d., 75 μm; resin, 3 μm; Thermo Scientific) and separated with a linear gradient of buffer B (84% acetonitrile and 0.1% FA) at a flow rate of 300 nl/min. The linear gradient was as follows: 0%–55% buffer B for 110 min, 55%–100% buffer B for 5 min, and a hold in 100% buffer B for 5 min.

The separated peptides were then examined via MS on a Q Exactive mass spectrometer (Thermo Fisher Scientific) in positive ion mode for 240 min. MS data was acquired using a data-dependent top 10 method, with only the most abundant precursor ions from the survey scan dynamically chosen (300–1800 m/z) for HCD fragmentation. The dynamic exclusion duration was 40.0 s. Survey scans were acquired at a resolution of 70,000 at m/z 200 and the resolution for the HCD spectra was set to 17,500 at m/z 200, with an isolation width of 2 m/z. The normalized collision energy was 30%, with an underfill ratio of 0.1%, which specifies the minimum percentage of the target value likely to be reached at the maximum fill time.

**Database searching of lysine-acetylated peptides and proteins**

First, the raw data files obtained from the MS analysis were processed using MaxQuant with an integrated Andromeda search engine (v.1.4.1.2) [[2](#_ENREF_2)]. The mass spectra were queried in Uniprot against the *A. hydrophila* ATCC7966 protein database, which contains 4,123 protein sequences (http:// [www.uniprot.org/](http://www.uniprot.org/)). The cleavage enzyme was specified as trypsin/P, with up to four missing cleavages, five charges, and five modifications per peptide permitted. Other specifications included carbamidomethylation of cysteine being set as a fixed modification and methionine oxidation, lysine acetylation, and N-terminal acylation were specified as variable modifications. Additionally, the mass error was set to 10 ppm for the precursor ions and 0.02 Da for fragment ions, with a minimum peptide length of seven amino acids. Furthermore, for peptides, proteins, and modification sites, the false discovery rate (FDR) threshold was set to 0.01. All other parameters in MaxQuant were set to the default parameters. The identified lysine acetylation sites with a localization probability >0.75 were selected. The raw mass data have been deposited to the ProteomeXchange database and are available with the accession number PXD012270.

**Bioinformatics analysis**

The identified acetylated proteins were then further examined by performing a GO annotation analysis using the UniProt-GOA database (<http://www.ebi.au.uk/GOA/>), with proteins classified into the main categories of biological process and molecular function. Protein pathways were annotated using the KEGG database, with the KAAS tool used first for protein analysis and annotation, followed by KEGG mapper, which maps the annotation results [[3](#_ENREF_3)]. Functional annotations for the protein domains were obtained using InterProScan coupled with the InterPro domain database [[4](#_ENREF_4)]. All the annotation results were statistically examined using a Fisher’s exact test (two-tailed test) and standard false discovery rate control methods were employed to correct for multiple-hypothesis testing. Any term with an adjusted *p*-value <0.05 was considered significant. Protein−protein interaction networks for the identified acetylated proteins were analyzed based on the STRING database and were visualized using Cytoscape [[5](#_ENREF_5)]. Finally, any overlap between acetylated peptides/proteins identified herein and the succinylated peptides/proteins identified in our previously study was identified.

**PTM proteomic analysis validation via Co-immunoprecipitation**

To further investigate the impact of acetylated lysine, several candidate proteins were analyzed by Co-IP and western blotting as previously described with slight modification [[6](#_ENREF_6)]. *A. hydrophila* cells pellets were obtained as described above and were resuspended in 5 ml of PBS prior to sonication on ice for a total of 30 min at 9s intervals. The whole-cell lysates were separated by centrifuging at 12,000 x g for 30 min at 4°C, and 1 ml of the obtained total protein was incubated with 50 μl of rabbit antisera overnight at 4°C, with pre-immune serum used as a negative control. Next, 50 μl of protein G agarose beads (Beyotime Biotechnology Shanghai, China) were added into the mixture, followed by an incubation for an additional 3–5 h at 4°C. The samples were then washed five times with cold PBS, and the proteins were eluted with 50 μl of 2 x SDS loading buffer. The Co-IP proteins were separated using SDS-PAGE, and detected using Western blotting.

**Western blotting**

Following SDS-PAGE, the electrophoresed proteins were transferred to a polyvinylidene difluoride membranes (PVDF; Millipore, Burlington, MA, USA) via a semi-dry electrophoretic transfer as described previously [[7](#_ENREF_7),[8](#_ENREF_8)]. The transfer was performed in 10x Transfer Buffer (Bio-Rad, Hercules, CA, USA) for 15 min at 25 V using a Trans-Blot Turbo Transfer System (Bio-Rad). The PVDF membranes were then blocked with 5% skim milk in PBST buffer (1 x PBS buffer containing 0.05% Tween-20; pH 7.4) or bovine serum albumin (BSA) in TBST buffer (20 mM Tris-HCl, 500 mM NaCl and 0.05% Tween-20; pH 7.6) and then incubated with primary antibodies, including anti-acetyl lysine (PTM Biolabs, Inc. Hangzhou, China). After rinsing three times with PBST or TBST for 10 min each time, the membranes were incubated with secondary antibodies. Protein bands were visualized using Clarity Western ECL substrate (Bio-Rad) and imaged with a ChemiDoc XRS+ system (Bio-Rad). Protein molecular weights were determined using a PageRuler^TM^ Plus Prestained Protein Ladder (Thermo Fisher Scientific).

**Constructing the *ΔluxS* and *ΔcobB* complement mutants**

The *luxS* and *cobB* knockout mutants, and the complementation strain carrying a pBBR1-MCS1::*luxS*-His-tagged vector were constructed in our laboratory [[9-11](#_ENREF_9)]. Firstly, the *luxS* and *cobB* gene depleted mutants were produced by the pRE112 suicide vector carrying the *sacB* gene. Approximate 500 base pairs (bp) of upstream and downstream flanking sequences of target genes were amplified from the genome DNA of *A. hydrophila* ATCC7966, and then were fused into the pRE112 vector by overlapping PCR producing the recombinant constructs. The constructs were introduced into *E. coli* MC1061(λpir) to elevate the transformation efficiency and subsequently transformed into *E. coli* strain S17-1(λpir) for quick propagation Next, the recombinant plasmids of *E. coli* S17-1were transferred into *A. hydrophila* via bacterial conjugation. Then, the first homologous recombination was performed to initially obtain single-crossover mutants of target genes on LB agar with ampicillin (100 µg/mL, Amp^R^) and chloramphenicol (30 µg/mL, Cm^R^). Subsequently, the gene depleted mutants were obtained through the second homologous recombination on LB agar containing 20 % (w/v) sucrose based on the *sacB* gene.

For site-directed mutations of *luxS*, the lysine (K, codon: AAG) was replaced with glutamate (E, codon: GAG), arginine (R, codon: AGG), and glutamine (Q, codon: CAG) at position 165 of the amino acid sequence by using a Fast Mutagenesis System Kit (TransGen Biotech, Beijing, China). The targeted point mutation plasmids, including pBBR1-MCS1::*luxS* K165E-His tag, pBBR1-MCS1::*luxS* K165R-His tag and pBBR1-MCS1::*luxS* K165Q-His tag, were amplified with *luxS*-K165E-F and R, *luxS*-K165R-F and R and *luxS*-K165Q-F and R primers using the supercoiled pBBR1-MCS1::*luxS*-His tag plasmid as the template. The PCR products were digested with DMT enzyme (DpnI restriction enzyme) from the kit and were subsequently transformed into DMT competent cells, with the recombinant cells sequenced for validation. Next, the targeted vectors were electroporated into the Δ*luxS* competent cells and the positive clones were further selected on LB agar with 30 μg/ml chloramphenicol. Similarly, the pBBR1-MCS1::*luxS*-His-tag vector was also transformed into Δ*cobB* competent cells, and complementary wild-type strain was used as a positive control. Additionally, all primers used in the current study are listed in **Table S2**.

**Pull-down assay**

His tag pull-downs were performed as previously described with some modifications [[12](#_ENREF_12)]. Complemented mutant colonies were incubated in 5 ml LB medium overnight at 30°C, and then diluted in 200 ml fresh LB medium at a ratio of 1:100 until an OD_600nm_ of 1.0 was reached. The cells were harvested, washed twice with saline, exposed to intermittent sonication in binding buffer [50 mM Tris-HCl (pH 8.0), 300 mM NaCl, and 5 mM imidazole], and centrifuged to remove cellular debris. The obtained lysates were then incubated with a Ni-NAT resin column overnight at 4°C. The bound proteins were washed five times with binding buffer and a buffer containing 50 mM Tris-HCl (pH 8.0), 300 mM NaCl, and 20 mM imidazole. The proteins were ultimately eluted with buffer containing 50 mM Tris-HCl (pH 8.0), 300 mM NaCl, and 300 mM imidazole. The eluted proteins were then analyzed by SDS-PAGE and Western blotting analysis with anti-acetyl lysine, anti-succinyl lysine (PTM Biolabs, Inc.), and anti-His tag (CW Biotech, Beijing, China) antibodies utilized.

**Assay for AI-2 activity in the *luxS* mutant strains**

The AI-2 levels following site-directed mutagenesis of the *luxS* gene were determined using the *Vibrio harveyi* BB170 bioluminescence reporter strain as previously described [[13](#_ENREF_13)]. Cell-free culture fluids were collected via centrifuging at 12,000 x g for 5 min and filtered (0.22 μm). The reporter strain was inoculated in AB medium and subcultivated in fresh medium overnight at 30°C. The samples were then diluted 1:5,000 with fresh AB medium and 20 μl of free culture supernatant was mixed with 180 μl of diluted *V. harveyi* BB170 and cultured in a black flat-bottom 96-well plate (Castor). The supernatant from the *V. harveyi* BB170 that was cultivated overnight was used as the positive control, while fresh AB medium was used as the negative control. The bioluminescence intensities were recorded hourly from 0–12 h using a SpectraMax i3x (Molecular Devices, Sunnyvale, CA, USA) at an absorbance of 490 nm at 30°C. AI-2 activity was quantified as a fold-change in relative luminescence units, that is, the ratio of luminescence produced in a sample relative to the positive control.

**Enzyme activity assay examining *in vitro* acetylated and succinylated LuxS**

The *luxS* gene and its derivatives were cloned into pET-32a vectors and overexpressed in *E. coli* BL21 (DE3) cells. The expression and purification procedures for the recombinant proteins were essentially conducted as a “pull-down assay”. Purified proteins were buffer-exchanged, with the buffer (50 mM Tris-HCl, 300 mM NaCl; pH 7.5) used to remove imidazole, using an Amicon Ultra 3K device (Millipore). To determine acetylation levels, purified proteins were incubated with freshly prepared potassium lithium salt (AcP; Sigma-Aldrich, St. Louis, MO, USA) at a final concentration of 20 mM in 150 mM Tris-HCl (pH 7.3), 10% glycerol, 10 mM MgCl_2_, and 150 mM NaCl. The samples were then incubated for 3 h at 37°C, and acetylation levels were detected via Western blotting using anti-acetyl lysine antibodies and the enzymatic activity of acetylated LuxS was determined by the following protocol [[14](#_ENREF_14),[15](#_ENREF_15)]. Meanwhile, the purified LuxS was also utilized to examine succinylation levels as previously described [[16](#_ENREF_16)]. First, the buffer that the purified proteins were solubilized in was replaced with 100 mM HEPES (pH 8.0), 0.8% octyl β-D-glucopyranoside, and 10% (vol/vol) glycerol. Next, 3 mM succinyl coenzyme A sodium salt (succinyl-CoA; Sigma) was mixed with 50 nM of purified protein in 100 mM HEPES (pH 7.4), 100 mM KCl, 10% (vol/vol) glycerol, 1 mM CoA, and 1 mM NAD, and incubated for 20 min at 27°C. Succinylation levels were also examined using Western blotting analysis and anti-succinyl lysine antibodies. Simultaneously, the enzyme activity of succinylated LuxS was estimated using the same method.

To investigate the enzymatic activity of LuxS and its derivatives, 2.3 μM of AcP-treated or untreated proteins were incubated with S-(5′-Adenosyl)-L-homocysteine (Sigma) at a final concentration of 1 mM in 250 μl of assay solution (100 mM Tris-HCl; pH 8.0) at 37°C for 35 min [[17](#_ENREF_17)]. Next, 750 μl of quenching solution [133 mM 5,50-dithiobis-2-nitrobenzoic acid (DTNB), 8 M guanidinium chloride, 100 mM potassium phosphate, 1 mM EDTA; pH 7.2] was added and incubated at 37°C for 30 min. Sample enzymatic activity levels were monitored using a SpectraMax i3x (Molecular Devices) at an absorbance of 412 nm.

**Co-culturing experiments**

As the AI-2 synthase, the LuxS enzyme encoded by the *luxS* gene is responsible for the AI-2 biosynthesis in quorum sensing, thereby influencing the bacterial cell-cell communication in response to population density. The adopted co-culture with *A. hydrophila* and *Vibrio alginolyticus* ATCC 33787 that is a major opportunistic pathogen in aquaculture and can be distinguished easily based on its specific colony morphology, was performed as previously described with several modifications [[18](#_ENREF_18)]. Briefly, pure overnight-cultures of *V. alginolyticus* ATCC 33787 (as a competitor) and *luxS* mutants (different participants) were subcultured in fresh LB medium and grown to an OD_600nm_ of 1.0 prior to combining. The *V. alginolyticus* inoculum was then mixed with an equal volume of each of the *luxS* mutant cultures, and subsequently diluted 1:10 with fresh medium. Co-cultures were cultivated with shaking for 12 h at 30°C. Due to the distinctly different colonial morphologies, colony-forming units for each the species were determined on LB agar plates after co-culturing. Moreover, the colonies for each specie were further confirmed by amplifying and sequencing the conserved 16S rDNA.

**Site-specific acetyllysine in recombinant LuxS protein**

To encode the N^ε^ - acetyllysine in LuxS protein at K165, a two-plasmid system was used in the current study as previously described with slightly modifications [[15](#_ENREF_15),[19-21](#_ENREF_19)]. In this system, the pET-21b vector was used to encode the proteins of interest, and the pTECH-MbAcK3RS(IPYE) plasmid could incorporate non-canonical amino acid and produce the acetyl lysine by encoding the orthogonal aaRS and the amber suppressor tRNA in response to the amber codon. Firstly, the *luxS* gene that installed an amber stop codon at K165 (AAG > TAG) was introduced into the pET-21b vector producing the C-terminal 6×His -tagged recombinant using the Fast Mutagenesis System Kit (TransGen Biotech, Beijing, China). Then, the amber mutated vector was transformed into the *E. coli* BL21 (DE3) with the pTECH-MbAcK3RS(IPYE) plasmid that encodes the MbAcK3RS(IPYE) and pylT and can be in response to the amber codon, and the positive clones were selected on LB agar with 100 μg /ml ampicillin and 30 μg/ml chloramphenicol. The amber mutated LuxS in *E. coli* BL21 (DE3) was overexpressed in LB media with ampicillin and chloramphenicol. At OD600 of 0.6-0.8, the culture was supplemented with 2 mM N-acetyllysine (Sigma-Aldrich) and 20 mM nicotinamide and incubated for 30 min at 37°C. Next, protein expression was continuously induced with the 1 mM isopropy-β-D-thiogalactoside (IPTG) at 16°C for 18h. The cells were harvested after induction and K165 – acetylated LuxS recombinant protein was purified based on the 6×His tag. The purified protein was used for enzyme activity assay and Western blotting analysis with anti-acetyl lysine antibody.

**References**

[1] Xie LX, Fang WJ, Deng WY, et al. Global profiling of lysine acetylation in human histoplasmosis pathogen *Histoplasma capsulatum*. Int J Biochem Cell Biol. 2016;73:1-10.

[2] Cox J, Neuhauser N, Michalski A, et al. Andromeda: a peptide search engine integrated into the MaxQuant environment. J Proteome Res. 2011;10(4):1794-1805.

[3] Moriya Y, Itoh M, Okuda S, et al. KAAS: an automatic genome annotation and pathway reconstruction server. Nucleic Acids Res. 2007;35(Web Server issue):W182-185.

[4] Zdobnov EM, Apweiler R. InterProScan – an integration platform for the signature-recognition methods in InterPro. Bioinformatics. 2001;17(9):847-848.

[5] Shannon P, Markiel A, Ozier O, et al. Cytoscape: a software environment for integrated models of biomolecular interaction networks. Genome Res. 2003;13(11):2498-2504.

[6] Lin XM, Yang MJ, Li H, et al. Decreased expression of LamB and Odp1 complex is crucial for antibiotic resistance in *Escherichia coli*. J Proteomics. 2014;98:244-253.

[7] Yang J, Zeng ZH, Yang MJ, et al. NaCl promotes antibiotic resistance by reducing redox states in *Vibrio alginolyticus*. Environ Microbiol. 2018;20(11):4022-4036.

[8] Su YB, Peng B, Li H, et al. Pyruvate cycle increases aminoglycoside efficacy and provides respiratory energy in bacteria. Pro Natl Acad Sci U S A. 2018;115(7):E1578-E1587.

[9] Yao ZJ, Sun LN, Wang YQ, et al. Quantitative proteomics reveals antibiotics resistance function of outer membrane proteins in *Aeromonas hydrophila*. Front Cell Infect Mi. 2018;8:390.

[10] Yao ZJ, Guo Z, Wang YQ, et al. Integrated succinylome and metabolome profiling reveals crucial role of S-ribosylhomocysteine lyase in quorum sensing and metabolism of *Aeromonas hydrophila*. Mol Cell Proteomics. 2019;18(2):200-215.

[11] Pang HY, Qiu MS, Zhao JM, et al. Construction of a *Vibrio alginolyticus hopPmaJ (hop)* mutant and evaluation of its potential as a live attenuated vaccine in orange-spotted grouper (*Epinephelus coioides*). Fish Shellfish Immunol. 2018;76:93-100.

[12] Lee KH, Kim MY, Kim DH, et al. Syntaxin 1A and receptor for activated C kinase interact with the N-terminal region of human dopamine transporter. Neurochem Res. 2004;29(7):1405-1409.

[13] Ju XY, Li JY, Zhu MJ, et al. Effect of the *luxS* gene on biofilm formation and antibiotic resistance by *Salmonella serovar Dublin*. Food Res Int. 2018;107:385-393.

[14] Weinert BT, Iesmantavicius V, Wagner SA, et al. Acetyl-phosphate is a critical determinant of lysine acetylation in *E. coli*. Mol Cell. 2013;51(2):265-272.

[15] Chen Z, Luo L, Chen RF, et al. Acetylome profiling reveals extensive lysine acetylation of the fatty acid metabolism pathway in the diatom *Phaeodactylum tricornutum*. Mol Cell Proteomics. 2018;17(3):399.

[16] Sadhukhan S, Liu XJ, Ryu D, et al. Metabolomics-assisted proteomics identifies succinylation and SIRT5 as important regulators of cardiac function. Pro Natl Acad Sci U S A. 2016;113(16):4320-4325.

[17] Zhao G, Wan W, Mansouri S, et al. Chemical synthesis of S-ribosyl-l-homocysteine and activity assay as a LuxS substrate. Bioorg Med Chem Lett. 2003;13(22):3897-3900.

[18] Smalley NE, An D, Parsek MR, et al. Quorum sensing protects *Pseudomonas aeruginosa* against cheating by other species in a laboratory coculture model. J Bacteriol. 2015;197(19):3154-3159.

[19] Neumann H, Hancock SM, Buning R, et al. A method for genetically installing site-specific acetylation in recombinant histones defines the effects of H3 K56 acetylation. Mol Cell. 2009;36(1):153-163.

[20] Brabham R, Fascione MA. Pyrrolysine amber stop-codon suppression: development and applications. Chembiochem. 2017;18(20):1973-1983.

[21] Bryson DI, Fan CG, Guo LT, et al. Continuous directed evolution of aminoacyl-tRNA synthetases. Nat Chem Biol. 2017;13(12):1253-1260.
