# Supplementary material for: Comprehensive analysis of the lysine acetylome in Aeromonas hydrophila reveals cross-talk between lysine acetylation and succinylation in LuxS
Source: Emerg Microbes Infect. 2019 Aug 26;8(1):1229–39. doi: 10.1080/22221751.2019.1656549 (PMC6735345; doi:10.1080/22221751.2019.1656549)
Supplement: Supplemental Material [file TEMI_A_1656549_SM0185.zip › SII Appendixv1.docx]

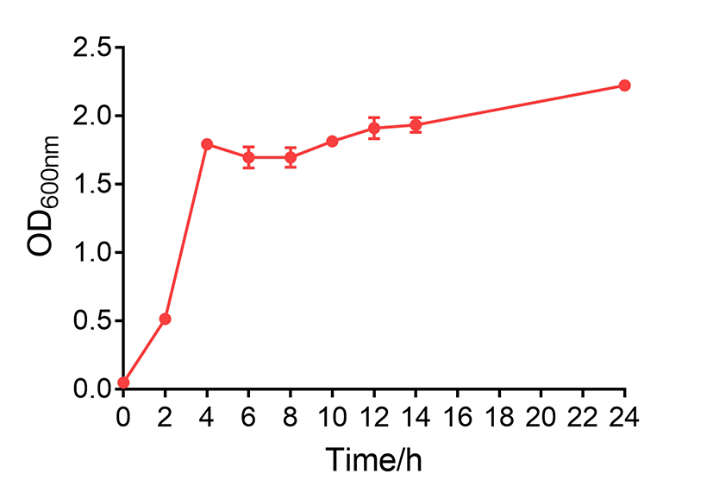


**Figure S1. The growth curve of *A. hydrophila* ATCC 7966.**


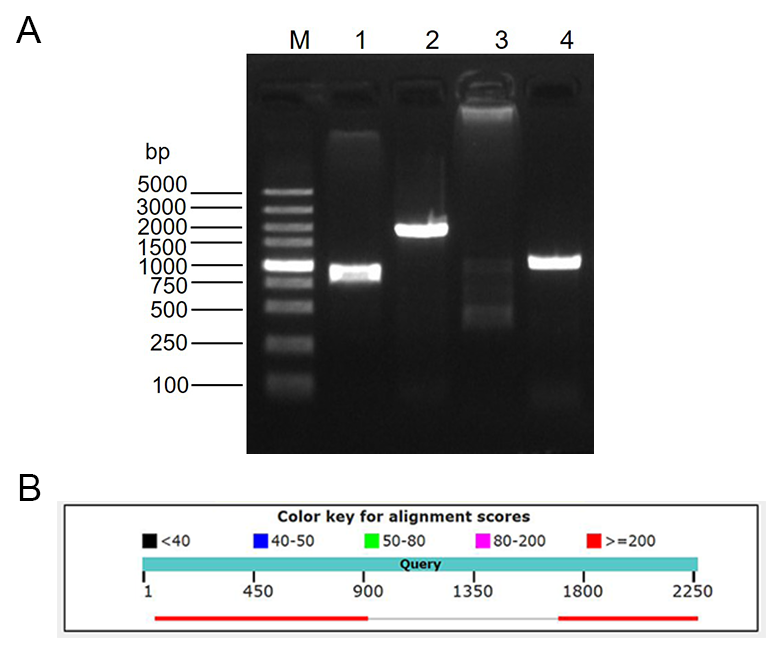


**Figure S2. Validation of the knockout mutant strain Δ*cobB*.**

A, The depleted mutant Δ*cobB* by colony PCR amplification. M: DL5000 marker; Lane 1 and 2, the fragment of genomic wild-type (*A. hydrophila* ATCC 7966) DNA amplified using P5/ P6 (774bp) and P7/P8 (2262bp), respectively; Lane 3 and 4, the fragment of genomic deletion mutant (Δ*cobB*) DNA amplified using P5/ P6 (undetectable) and P7/P8 (1488bp), respectively. P5/ P6 is forward/ reverse primer of *cobB* gene sequence, respectively, and P7/P8 is located at the front of the upstream/ downstream sequence of the *cobB* gene, respectively. The primer pairs for constructing the *cobB* knockout strai in this study were listed in **Table S2**; B, The confirmation of Δ*cobB* strain by DNA sequencing with primer pairs P7/P8. The sequencing result is aligned with wild type by BLAST online software and showed that the target gene was absent.
