# Supplementary material for: Comprehensive analysis of the lysine acetylome in Aeromonas hydrophila reveals cross-talk between lysine acetylation and succinylation in LuxS
Source: Emerg Microbes Infect. 2019 Aug 26;8(1):1229–39. doi: 10.1080/22221751.2019.1656549 (PMC6735345; doi:10.1080/22221751.2019.1656549)
Supplement: Supplemental Material [file TEMI_A_1656549_SM0185.zip › Table S2v1.docx]

**Supplementary Table S2**

| Primer | Sequence (5’→3’) | Description |
| --- | --- | --- |
| pBBR1-*luxS*-F | gtcgacggtatcgataagcttgTGAAACCTGGGCTGACTACCA | Forward primer for complementing *luxS* gene |
| pBBR1-*luxS*-R | cgctctagaactagtggatccGAGGCTTTTCAGCTTCTCTTCCG | Reverse primer for complementing *luxS* gene |
| *luxS*-K165E-F | CTGCCGGAAGAGGAGCTGAAAAGCCTCT | Forward primer for K165E of LuxS |
| *luxS*-K165E-R | CCTCTTCCGGCAGGGCCAGCTCGTCATT | Reverse primer for K165E of LuxS |
| *luxS*-K165R-F | CTGCCGGAAGAGAGGCTGAAAAGCCTCT | Forward primer for K165R of LuxS |
| *luxS*-K165R-R | CCTCTTCCGGCAGGGCCAGCTCGTCAT | Reverse primer for K165R of LuxS |
| *luxS*-K165Q-F | CTGCCGGAAGAGCAGCTGAAAAGCCTCT | Forward primer for K165Q of LuxS |
| *luxS*-K165Q-R | CCTCTTCCGGCAGGGCCAGCTCGTCATT | Reverse primer for K165Q of LuxS |
| *cobB*-P1 | cgatcccaagcttcttctagaCTTGGGATAGGTGGTGAACGG | Forward primer of upstream sequence |
| *cobB*-P2 | ggaatcagtcGCTATTGTGCGACTGGACAGTTG | Reverse primer of upstream sequence |
| *cobB*-P3 | gcacaatagcGACTGATTCCCTCTGGCTACTTCT | Forward primer of downstream sequence |
| *cobB*-P4 | catgaattcccgggagagctcGCGAGCAGAGCGTCTACCTG | Reverse primer downstream sequence |
| *cobB*-P5 | CATGGTGCAGTCAGCGAAACAC | Forward primer of *cobB* gene sequence |
| *cobB*-P6 | ACTGTCCAGTCGCACAATAGCTC | Reverse primer of *cobB* gene sequence |
| *cobB*-P7 | TGCTGGACAACGATCAGCACAT | Locating at the front of the upstream sequence |
| *cobB*-P8 | CATCAATCGACGATCTCGATGC | Locating at the back of the downstream sequence |
